# Aqua­chloridobis(2-eth­oxy-6-formyl­phenolato-κ^2^ *O* ^1^,*O* ^6^)chromium(III) acetonitrile hemisolvate

**DOI:** 10.1107/S1600536811037275

**Published:** 2011-09-17

**Authors:** Safoora Ghelenji, Hadi Kargar, Zahra Sharafi, Reza Kia

**Affiliations:** a Department of Chemistry, North Tehran Branch, Islamic Azad University, Tehran, Iran; b Chemistry Department, Payame Noor University, Tehran 19395-4697, I. R. of Iran; c Department of Chemistry, Marvdasht Branch, Islamic Azad University, Marvdasht, Iran; d X-ray Crystallography Laboratory, Plasma Physics Research Center, Science and Research Branch, Islamic Azad University, Tehran, Iran; e Department of Chemistry, Science and Research Branch, Islamic Azad University, Tehran, Iran

## Abstract

In the mononuclear complex mol­ecule of the title compound, [Cr(C_9_H_9_O_3_)_2_Cl(H_2_O)]·0.5CH_3_CN, the Cr^III^ atom displays an elongated octa­hedral coordination geometry. The dihedral angle between the benzene rings is 12.27 (11)°. Adjacent complex mol­ecules are linked into dimers by O—H⋯O hydrogen bonds, generating rings of *R*_1_^2^(6) and *R*_1_^2^(5) graph-set motifs, and by aromatic π–π stacking inter­actions, with a centroid–centroid distance of 3.812 (2) Å. The crystal packing is further stabilized by inter­molecular C—H⋯N hydrogen bonds. The C and N atoms of the acetonitrile solvent mol­ecule are located on a crystallographic twofold axis.

## Related literature

For details of hydrogen-bond motifs, see: Bernstein *et al.* (1995 ▶). For the structures of tetra­dentate Schiff bases synthesized by our group, see: Kargar *et al.* (2009 ▶, 2010 ▶).
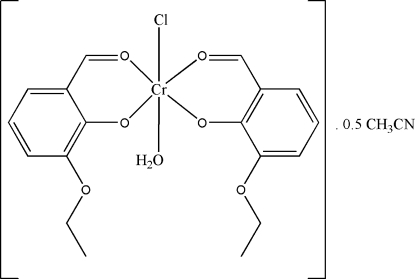


## Experimental

### 

#### Crystal data

[Cr(C_9_H_9_O_3_)_2_Cl(H_2_O)]·0.5C_2_H_3_N
*M* *_r_* = 456.32
Monoclinic, 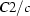

*a* = 19.292 (3) Å
*b* = 10.1211 (10) Å
*c* = 20.953 (3) Å
β = 91.824 (11)°
*V* = 4089.1 (10) Å^3^
*Z* = 8
Mo *K*α radiation
μ = 0.73 mm^−1^
*T* = 291 K
0.25 × 0.15 × 0.12 mm

#### Data collection

Stoe IPDS 2T Image Plate diffractometer
Absorption correction: multi-scan [*MULABS* (Blessing, 1995 ▶) in *PLATON* (Spek, 2009 ▶)] *T*_min_ = 0.901, *T*_max_ = 1.000
9374 measured reflections
4371 independent reflections
2108 reflections with *I* > 2σ(*I*)
*R*_int_ = 0.070

#### Refinement

*R*[*F* ^2^ > 2σ(*F* ^2^)] = 0.051
*wR*(*F* ^2^) = 0.078
*S* = 0.80
4371 reflections
261 parameters
H-atom parameters constrained
Δρ_max_ = 0.27 e Å^−3^
Δρ_min_ = −0.33 e Å^−3^

### 

Data collection: *X-AREA* (Stoe & Cie, 2009 ▶); cell refinement: *X-AREA*; data reduction: *X-AREA*; program(s) used to solve structure: *SHELXTL* (Sheldrick, 2008 ▶); program(s) used to refine structure: *SHELXTL*; molecular graphics: *SHELXTL*; software used to prepare material for publication: *SHELXTL* and *PLATON* (Spek, 2009 ▶).

## Supplementary Material

Crystal structure: contains datablock(s) global, I. DOI: 10.1107/S1600536811037275/rz2636sup1.cif

Structure factors: contains datablock(s) I. DOI: 10.1107/S1600536811037275/rz2636Isup2.hkl

Additional supplementary materials:  crystallographic information; 3D view; checkCIF report

## Figures and Tables

**Table 1:** Hydrogen-bond geometry (Å, °)

*D*—H⋯*A*	*D*—H	H⋯*A*	*D*⋯*A*	*D*—H⋯*A*
O1*W*—H1*W*1⋯O1^i^	0.85	2.10	2.826 (3)	143
O1*W*—H1*W*1⋯O5^i^	0.85	2.28	3.007 (4)	144
O1*W*—H2*W*1⋯O3^i^	0.85	2.22	2.813 (3)	127
O1*W*—H2*W*1⋯O6^i^	0.85	2.14	2.940 (4)	158
C7—H7*A*⋯N1^ii^	0.93	2.62	3.171 (4)	119

Symmetry codes: (i) 
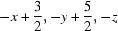
; (ii) 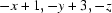.

**Fig. 1.**
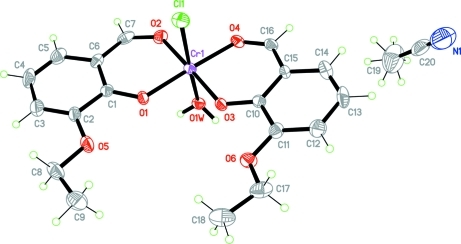
The molecular structure of the title compound, showing 30% probability displacement ellipsoids. The acetonitrile solvent is disordered about a crystallographic twofold rotation axis.

**Fig. 2.**
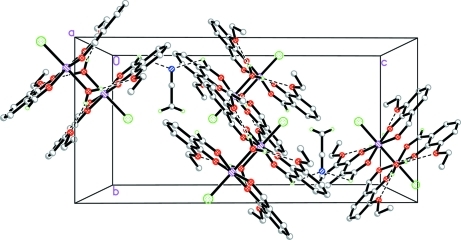
Crystal packing of the title compound viewed down the a axis. Intermolecular hydrogen interactions are shown as dashed lines. H atoms not involved in hydrogen bonding are omitted for clarity.

## supplementary crystallographic information

### Comment

As part of our ongoing study of potential tetradenate Schiff bases (Kargar *et al.*, 2009; Kargar *et al.* 2010) derived from different substituted salicylaldehydes, we have determined the crystal structure of the title compound, which was obtained by the reaction of chromium(III) chloride hexahydrate with 3-ethoxysalicylaldehyde in acetonitrile.

The asymmetric unit of the title compound, Fig. 1, comprises one mononuclear complex molecule and one half of an acetonitrile solvent molecule, whose C and N atoms are located on a crystallographic twofold axis. In the complex molecule, the metal atom displays an elongated octahedral coordination geometry. The dihedral angles between the substituted benzene rings is 12.27 (11)°. Strong intermolecular O—H···O hydrogen bonds (Table 1) link adjacent complex molecules into dimers, generating rings of *R^2^_1_(6)* and *R^2^_1_(5)* graph set motifs (Bernstein *et al.*, 1995). In the dimers, aromatic π–π stacking interactions with centroid-to-centroid distance of 3.812 (2) Å are observed (Table 1). The crystal packing (Fig. 2) is further stabilized by C—H···N hydrogen bonds involving the acetonitrile molecule.

### Experimental

The title compound was synthesized by adding 3-ethoxy-salicylaldehyde (4 mmol) to a solution of CrCl_3_. 6H_2_O (2 mmol) in acetonitrile (50 ml). The mixture was refluxed with stirring for 3 h. The resultant dark-green solution was filtered and single crystals suitable for *X*-ray structure determination were grown from the solution by slow evaporation of the solvent at room temperature over several days.

### Refinement

H atoms of the water molecule were located in a difference Fourier map, first restraied to a distance of 0.85 (1)Å and then constrained to refine with the parent atom with *U*_iso_(H) = 1.5 *U*_eq_(O). The remaining H atoms were positioned geometrically with C—H = 0.93 – 0.96 Å and included in a riding model approximation with *U*_iso_ (H) = 1.2 or 1.5 *U*_eq_ (C). A rotating group model was used for the methyl groups.

### Crystal data

**Table:**

[Cr(C_9_H_9_O_3_)_2_Cl(H_2_O)]·0.5C_2_H_3_N	*F*(000) = 1888
*M**_r_* = 456.32	*D*_x_ = 1.482 Mg m^−^^3^
Monoclinic, *C*2/*c*	Mo *K*α radiation, λ = 0.71073 Å
Hall symbol: -C 2yc	Cell parameters from 1749 reflections
*a* = 19.292 (3) Å	θ = 2.2–29.5°
*b* = 10.1211 (10) Å	µ = 0.73 mm^−^^1^
*c* = 20.953 (3) Å	*T* = 291 K
β = 91.824 (11)°	Block, dark-green
*V* = 4089.1 (10) Å^3^	0.25 × 0.15 × 0.12 mm
*Z* = 8	

### Data collection

**Table:**

Stoe IPDS 2T Image Plate diffractometer	4371 independent reflections
Radiation source: fine-focus sealed tube	2108 reflections with *I* > 2σ(*I*)
graphite	*R*_int_ = 0.070
Detector resolution: 0.15 mm pixels mm^-1^	θ_max_ = 27.0°, θ_min_ = 1.9°
ω scans	*h* = −24→24
Absorption correction: multi-scan [*MULABS* (Blessing, 1995) in *PLATON* (Spek, 2009)]	*k* = −11→12
*T*_min_ = 0.901, *T*_max_ = 1.000	*l* = −23→26
9374 measured reflections	

### Refinement

**Table:**

Refinement on *F*^2^	Primary atom site location: structure-invariant direct methods
Least-squares matrix: full	Secondary atom site location: difference Fourier map
*R*[*F*^2^ > 2σ(*F*^2^)] = 0.051	Hydrogen site location: inferred from neighbouring sites
*wR*(*F*^2^) = 0.078	H-atom parameters constrained
*S* = 0.80	*w* = 1/[σ^2^(*F*_o_^2^) + (0.0193*P*)^2^] where *P* = (*F*_o_^2^ + 2*F*_c_^2^)/3
4371 reflections	(Δ/σ)_max_ = 0.001
261 parameters	Δρ_max_ = 0.27 e Å^−^^3^
0 restraints	Δρ_min_ = −0.33 e Å^−^^3^

### Special details

**Table:**

Geometry. All e.s.d.'s (except the e.s.d. in the dihedral angle between two l.s. planes) are estimated using the full covariance matrix. The cell e.s.d.'s are taken into account individually in the estimation of e.s.d.'s in distances, angles and torsion angles; correlations between e.s.d.'s in cell parameters are only used when they are defined by crystal symmetry. An approximate (isotropic) treatment of cell e.s.d.'s is used for estimating e.s.d.'s involving l.s. planes.
Refinement. Refinement of *F*^2^ against ALL reflections. The weighted *R*-factor *wR* and goodness of fit *S* are based on *F*^2^, conventional *R*-factors *R* are based on *F*, with *F* set to zero for negative *F*^2^. The threshold expression of *F*^2^ > σ(*F*^2^) is used only for calculating *R*-factors(gt) *etc*. and is not relevant to the choice of reflections for refinement. *R*-factors based on *F*^2^ are statistically about twice as large as those based on *F*, and *R*- factors based on ALL data will be even larger.

### Fractional atomic coordinates and isotropic or equivalent isotropic displacement parameters (Å^2^)

**Table:**

	*x*	*y*	*z*	*U*_iso_*/*U*_eq_	Occ. (<1)
Cr1	0.64593 (2)	1.32691 (7)	0.04499 (3)	0.03258 (17)	
Cl1	0.61724 (4)	1.48966 (12)	0.11688 (5)	0.0500 (3)	
O1	0.72440 (10)	1.2660 (3)	0.09641 (12)	0.0352 (7)	
O2	0.58449 (11)	1.2011 (3)	0.08794 (13)	0.0421 (8)	
O3	0.70720 (10)	1.4377 (3)	−0.00154 (12)	0.0343 (7)	
O4	0.56652 (10)	1.3822 (3)	−0.01146 (13)	0.0395 (7)	
O5	0.84236 (11)	1.2384 (3)	0.15669 (13)	0.0506 (8)	
O6	0.80992 (11)	1.5786 (3)	−0.04008 (14)	0.0460 (8)	
C1	0.72533 (16)	1.1870 (4)	0.14529 (18)	0.0325 (9)	
C2	0.78879 (17)	1.1670 (4)	0.18084 (19)	0.0386 (10)	
C3	0.7924 (2)	1.0838 (5)	0.2312 (2)	0.0556 (13)	
H3A	0.8347	1.0717	0.2528	0.067*	
C4	0.7343 (2)	1.0156 (5)	0.2516 (2)	0.0632 (14)	
H4A	0.7378	0.9601	0.2870	0.076*	
C5	0.6731 (2)	1.0311 (5)	0.2196 (2)	0.0543 (13)	
H5A	0.6345	0.9839	0.2323	0.065*	
C6	0.66667 (19)	1.1183 (4)	0.16681 (19)	0.0385 (10)	
C7	0.60077 (19)	1.1291 (4)	0.1337 (2)	0.0455 (12)	
H7A	0.5658	1.0750	0.1485	0.055*	
C8	0.90836 (18)	1.2297 (5)	0.1897 (2)	0.0581 (14)	
H8A	0.9267	1.1407	0.1870	0.070*	
H8B	0.9039	1.2522	0.2344	0.070*	
C9	0.95559 (18)	1.3257 (6)	0.1581 (2)	0.0790 (17)	
H9A	1.0012	1.3198	0.1775	0.118*	
H9B	0.9381	1.4138	0.1630	0.118*	
H9C	0.9576	1.3050	0.1135	0.118*	
C10	0.69152 (17)	1.5300 (4)	−0.04271 (19)	0.0330 (10)	
C11	0.74590 (19)	1.6113 (4)	−0.0648 (2)	0.0415 (11)	
C12	0.7323 (2)	1.7116 (5)	−0.1067 (2)	0.0640 (15)	
H12A	0.7684	1.7646	−0.1200	0.077*	
C13	0.6644 (2)	1.7360 (5)	−0.1301 (2)	0.0764 (17)	
H13A	0.6555	1.8044	−0.1589	0.092*	
C14	0.6121 (2)	1.6586 (5)	−0.1102 (2)	0.0625 (14)	
H14A	0.5671	1.6753	−0.1255	0.075*	
C15	0.62361 (17)	1.5546 (4)	−0.06753 (19)	0.0381 (11)	
C16	0.56650 (17)	1.4756 (5)	−0.04983 (19)	0.0400 (11)	
H16A	0.5239	1.4965	−0.0693	0.048*	
C17	0.86615 (18)	1.6679 (5)	−0.0508 (2)	0.0595 (13)	
H17A	0.8541	1.7563	−0.0371	0.071*	
H17B	0.8761	1.6707	−0.0958	0.071*	
C18	0.92803 (19)	1.6186 (5)	−0.0130 (2)	0.0718 (17)	
H18A	0.9666	1.6765	−0.0192	0.108*	
H18B	0.9395	1.5312	−0.0269	0.108*	
H18C	0.9176	1.6166	0.0315	0.108*	
O1W	0.66200 (9)	1.1806 (3)	−0.01853 (11)	0.0375 (7)	
H1W1	0.6804	1.1980	−0.0537	0.056*	
H2W1	0.6804	1.1082	−0.0071	0.056*	
N1	0.5000	1.8521 (9)	−0.2500	0.123 (3)	
C19	0.5000	1.5944 (10)	−0.2500	0.137 (4)	
H19A	0.5241	1.5628	−0.2864	0.206*	0.50
H19B	0.4531	1.5628	−0.2520	0.206*	0.50
H19C	0.5229	1.5628	−0.2116	0.206*	0.50
C20	0.5000	1.7396 (12)	−0.2500	0.076 (3)	

### Atomic displacement parameters (Å^2^)

**Table:**

	*U*^11^	*U*^22^	*U*^33^	*U*^12^	*U*^13^	*U*^23^
Cr1	0.0213 (2)	0.0403 (4)	0.0361 (4)	−0.0020 (3)	−0.0004 (2)	0.0045 (4)
Cl1	0.0440 (5)	0.0546 (8)	0.0511 (7)	0.0016 (5)	−0.0026 (5)	−0.0124 (7)
O1	0.0267 (11)	0.0417 (18)	0.0369 (17)	−0.0002 (11)	−0.0029 (11)	0.0143 (15)
O2	0.0327 (13)	0.050 (2)	0.0435 (18)	−0.0103 (13)	0.0052 (13)	0.0062 (17)
O3	0.0236 (11)	0.0380 (18)	0.0412 (17)	0.0020 (11)	−0.0011 (11)	0.0118 (15)
O4	0.0241 (12)	0.052 (2)	0.0426 (18)	0.0052 (12)	−0.0045 (12)	0.0019 (16)
O5	0.0360 (13)	0.067 (2)	0.0477 (18)	0.0003 (13)	−0.0122 (13)	0.0142 (18)
O6	0.0334 (13)	0.0396 (18)	0.065 (2)	−0.0062 (12)	0.0060 (13)	0.0103 (17)
C1	0.0390 (18)	0.030 (3)	0.029 (2)	0.0044 (19)	0.0030 (17)	−0.005 (2)
C2	0.0449 (19)	0.038 (3)	0.033 (2)	0.007 (2)	−0.0006 (18)	0.003 (2)
C3	0.064 (3)	0.058 (3)	0.045 (3)	0.015 (2)	−0.001 (2)	0.009 (3)
C4	0.098 (4)	0.049 (3)	0.042 (3)	0.017 (3)	0.004 (3)	0.021 (3)
C5	0.071 (3)	0.042 (3)	0.050 (3)	−0.006 (2)	0.018 (2)	0.008 (3)
C6	0.050 (2)	0.037 (3)	0.029 (2)	−0.0024 (19)	0.008 (2)	−0.001 (2)
C7	0.047 (2)	0.044 (3)	0.047 (3)	−0.017 (2)	0.021 (2)	−0.003 (3)
C8	0.047 (2)	0.069 (4)	0.057 (3)	0.013 (2)	−0.018 (2)	−0.015 (3)
C9	0.043 (2)	0.113 (5)	0.080 (4)	−0.012 (3)	−0.011 (2)	0.000 (4)
C10	0.0368 (19)	0.029 (2)	0.033 (2)	0.0052 (17)	0.0041 (18)	−0.003 (2)
C11	0.048 (2)	0.032 (3)	0.044 (3)	−0.0016 (19)	0.005 (2)	0.004 (2)
C12	0.064 (3)	0.054 (4)	0.075 (4)	0.004 (2)	0.017 (3)	0.020 (3)
C13	0.083 (3)	0.065 (4)	0.081 (4)	0.015 (3)	0.003 (3)	0.047 (3)
C14	0.061 (3)	0.066 (4)	0.059 (3)	0.017 (3)	−0.011 (2)	0.015 (3)
C15	0.039 (2)	0.044 (3)	0.031 (2)	0.0149 (18)	−0.0003 (18)	0.009 (2)
C16	0.0324 (19)	0.056 (3)	0.031 (3)	0.013 (2)	−0.0046 (18)	−0.008 (3)
C17	0.055 (2)	0.041 (3)	0.084 (4)	−0.012 (2)	0.025 (2)	−0.007 (3)
C18	0.045 (2)	0.072 (4)	0.098 (4)	−0.022 (2)	0.006 (3)	−0.023 (3)
O1W	0.0300 (11)	0.0395 (17)	0.0432 (16)	0.0016 (12)	0.0049 (11)	0.0037 (16)
N1	0.144 (6)	0.099 (8)	0.129 (8)	0.000	0.057 (5)	0.000
C19	0.216 (11)	0.097 (10)	0.100 (9)	0.000	0.009 (7)	0.000
C20	0.078 (5)	0.107 (8)	0.043 (5)	0.000	0.023 (4)	0.000

### Geometric parameters (Å, °)

**Table:**

Cr1—O3	1.918 (2)	C9—H9A	0.9600
Cr1—O1	1.931 (2)	C9—H9B	0.9600
Cr1—O2	1.976 (3)	C9—H9C	0.9600
Cr1—O4	1.986 (3)	C10—C15	1.416 (5)
Cr1—O1W	2.021 (3)	C10—C11	1.422 (5)
Cr1—Cl1	2.3112 (13)	C11—C12	1.361 (6)
O1—C1	1.299 (4)	C12—C13	1.406 (6)
O2—C7	1.237 (5)	C12—H12A	0.9300
O3—C10	1.301 (4)	C13—C14	1.354 (6)
O4—C16	1.242 (4)	C13—H13A	0.9300
O5—C2	1.371 (4)	C14—C15	1.395 (6)
O5—C8	1.433 (4)	C14—H14A	0.9300
O6—C11	1.365 (4)	C15—C16	1.420 (5)
O6—C17	1.435 (4)	C16—H16A	0.9300
C1—C6	1.414 (5)	C17—C18	1.497 (6)
C1—C2	1.427 (5)	C17—H17A	0.9700
C2—C3	1.349 (5)	C17—H17B	0.9700
C3—C4	1.395 (5)	C18—H18A	0.9600
C3—H3A	0.9300	C18—H18B	0.9600
C4—C5	1.349 (6)	C18—H18C	0.9600
C4—H4A	0.9300	O1W—H1W1	0.8475
C5—C6	1.418 (6)	O1W—H2W1	0.8459
C5—H5A	0.9300	N1—C20	1.138 (11)
C6—C7	1.433 (5)	C19—C20	1.470 (12)
C7—H7A	0.9300	C19—H19A	0.9600
C8—C9	1.501 (6)	C19—H19B	0.9600
C8—H8A	0.9700	C19—H19C	0.9600
C8—H8B	0.9700		
			
O3—Cr1—O1	89.17 (10)	C8—C9—H9B	109.5
O3—Cr1—O2	175.31 (12)	H9A—C9—H9B	109.5
O1—Cr1—O2	90.64 (11)	C8—C9—H9C	109.5
O3—Cr1—O4	90.51 (11)	H9A—C9—H9C	109.5
O1—Cr1—O4	176.86 (12)	H9B—C9—H9C	109.5
O2—Cr1—O4	89.43 (11)	O3—C10—C15	124.2 (3)
O3—Cr1—O1W	89.06 (10)	O3—C10—C11	118.2 (3)
O1—Cr1—O1W	89.98 (10)	C15—C10—C11	117.6 (4)
O2—Cr1—O1W	86.25 (10)	C12—C11—O6	125.4 (4)
O4—Cr1—O1W	86.89 (10)	C12—C11—C10	120.8 (4)
O3—Cr1—Cl1	94.52 (9)	O6—C11—C10	113.9 (4)
O1—Cr1—Cl1	93.64 (9)	C11—C12—C13	121.0 (4)
O2—Cr1—Cl1	90.16 (8)	C11—C12—H12A	119.5
O4—Cr1—Cl1	89.50 (8)	C13—C12—H12A	119.5
O1W—Cr1—Cl1	174.93 (6)	C14—C13—C12	119.1 (5)
C1—O1—Cr1	128.9 (2)	C14—C13—H13A	120.5
C7—O2—Cr1	126.3 (2)	C12—C13—H13A	120.5
C10—O3—Cr1	128.5 (2)	C13—C14—C15	121.9 (4)
C16—O4—Cr1	125.8 (2)	C13—C14—H14A	119.0
C2—O5—C8	117.3 (3)	C15—C14—H14A	119.0
C11—O6—C17	117.9 (3)	C14—C15—C10	119.7 (4)
O1—C1—C6	124.3 (3)	C14—C15—C16	118.9 (4)
O1—C1—C2	119.3 (3)	C10—C15—C16	121.5 (4)
C6—C1—C2	116.5 (4)	O4—C16—C15	127.9 (4)
C3—C2—O5	126.6 (4)	O4—C16—H16A	116.1
C3—C2—C1	121.2 (4)	C15—C16—H16A	116.1
O5—C2—C1	112.1 (3)	O6—C17—C18	107.5 (4)
C2—C3—C4	121.8 (4)	O6—C17—H17A	110.2
C2—C3—H3A	119.1	C18—C17—H17A	110.2
C4—C3—H3A	119.1	O6—C17—H17B	110.2
C5—C4—C3	119.3 (4)	C18—C17—H17B	110.2
C5—C4—H4A	120.4	H17A—C17—H17B	108.5
C3—C4—H4A	120.4	C17—C18—H18A	109.5
C4—C5—C6	120.9 (4)	C17—C18—H18B	109.5
C4—C5—H5A	119.6	H18A—C18—H18B	109.5
C6—C5—H5A	119.6	C17—C18—H18C	109.5
C1—C6—C5	120.3 (4)	H18A—C18—H18C	109.5
C1—C6—C7	121.0 (4)	H18B—C18—H18C	109.5
C5—C6—C7	118.6 (4)	Cr1—O1W—H1W1	119.9
O2—C7—C6	128.3 (4)	Cr1—O1W—H2W1	121.3
O2—C7—H7A	115.9	H1W1—O1W—H2W1	104.1
C6—C7—H7A	115.9	C20—C19—H19A	109.5
O5—C8—C9	106.8 (4)	C20—C19—H19B	109.5
O5—C8—H8A	110.4	H19A—C19—H19B	109.5
C9—C8—H8A	110.4	C20—C19—H19C	109.5
O5—C8—H8B	110.4	H19A—C19—H19C	109.5
C9—C8—H8B	110.4	H19B—C19—H19C	109.5
H8A—C8—H8B	108.6	N1—C20—C19	180.000 (5)
C8—C9—H9A	109.5		
			
O3—Cr1—O1—C1	175.8 (3)	O1—C1—C6—C7	1.9 (6)
O2—Cr1—O1—C1	−8.9 (3)	C2—C1—C6—C7	−178.6 (4)
O1W—Cr1—O1—C1	−95.1 (3)	C4—C5—C6—C1	2.4 (6)
Cl1—Cr1—O1—C1	81.3 (3)	C4—C5—C6—C7	178.8 (4)
O1—Cr1—O2—C7	5.5 (3)	Cr1—O2—C7—C6	0.1 (6)
O4—Cr1—O2—C7	−177.6 (3)	C1—C6—C7—O2	−5.5 (6)
O1W—Cr1—O2—C7	95.5 (3)	C5—C6—C7—O2	178.2 (4)
Cl1—Cr1—O2—C7	−88.1 (3)	C2—O5—C8—C9	−174.6 (3)
O1—Cr1—O3—C10	−169.9 (3)	Cr1—O3—C10—C15	−8.8 (5)
O4—Cr1—O3—C10	13.2 (3)	Cr1—O3—C10—C11	171.7 (3)
O1W—Cr1—O3—C10	100.1 (3)	C17—O6—C11—C12	10.1 (6)
Cl1—Cr1—O3—C10	−76.3 (3)	C17—O6—C11—C10	−169.6 (3)
O3—Cr1—O4—C16	−12.4 (3)	O3—C10—C11—C12	−178.4 (4)
O2—Cr1—O4—C16	172.3 (3)	C15—C10—C11—C12	2.1 (6)
O1W—Cr1—O4—C16	−101.4 (3)	O3—C10—C11—O6	1.4 (5)
Cl1—Cr1—O4—C16	82.1 (3)	C15—C10—C11—O6	−178.1 (3)
Cr1—O1—C1—C6	6.6 (5)	O6—C11—C12—C13	179.1 (4)
Cr1—O1—C1—C2	−172.9 (3)	C10—C11—C12—C13	−1.2 (7)
C8—O5—C2—C3	−3.3 (6)	C11—C12—C13—C14	0.3 (8)
C8—O5—C2—C1	178.0 (3)	C12—C13—C14—C15	−0.5 (8)
O1—C1—C2—C3	−178.7 (4)	C13—C14—C15—C10	1.5 (7)
C6—C1—C2—C3	1.7 (6)	C13—C14—C15—C16	−178.6 (5)
O1—C1—C2—O5	0.0 (5)	O3—C10—C15—C14	178.2 (4)
C6—C1—C2—O5	−179.5 (3)	C11—C10—C15—C14	−2.3 (6)
O5—C2—C3—C4	−179.8 (4)	O3—C10—C15—C16	−1.6 (6)
C1—C2—C3—C4	−1.2 (7)	C11—C10—C15—C16	177.9 (4)
C2—C3—C4—C5	1.2 (7)	Cr1—O4—C16—C15	7.5 (6)
C3—C4—C5—C6	−1.8 (7)	C14—C15—C16—O4	−177.8 (4)
O1—C1—C6—C5	178.2 (4)	C10—C15—C16—O4	2.0 (6)
C2—C1—C6—C5	−2.3 (5)	C11—O6—C17—C18	173.5 (4)

### Hydrogen-bond geometry (Å, °)

**Table:**

*D*—H···*A*	*D*—H	H···*A*	*D*···*A*	*D*—H···*A*
O1W—H1W1···O1^i^	0.85	2.10	2.826 (3)	143.
O1W—H1W1···O5^i^	0.85	2.28	3.007 (4)	144.
O1W—H2W1···O3^i^	0.85	2.22	2.813 (3)	127.
O1W—H2W1···O6^i^	0.85	2.14	2.940 (4)	158.
C7—H7A···N1^ii^	0.93	2.62	3.171 (4)	119
C19—H19A···Cl1^iii^	0.96	2.80	3.745 (3)	167

Symmetry codes: (i) −*x*+3/2, −*y*+5/2, −*z*; (ii) −*x*+1, −*y*+3, −*z*; (iii) *x*, −*y*+3, *z*−1/2.

## References

[bb1] Bernstein, J., Davis, R. E., Shimoni, L. & Chang, N.-L. (1995). *Angew. Chem. Int. Ed. Engl.* **34**, 1555–1573.

[bb2] Blessing, R. H. (1995). *Acta Cryst.* A**51**, 33–38. 10.1107/s0108767394005726 7702794

[bb3] Kargar, H., Kia, R., Jamshidvand, A. & Fun, H.-K. (2009). *Acta Cryst.* E**65**, o776–o777. 10.1107/S1600536809008903 PMC2968825 21582502

[bb4] Kargar, H., Kia, R., Ullah Khan, I. & Sahraei, A. (2010). *Acta Cryst.* E**66**, o539. 10.1107/S1600536810002916 PMC2983671 21580310

[bb5] Sheldrick, G. M. (2008). *Acta Cryst.* A**64**, 112–122. 10.1107/S0108767307043930 18156677

[bb6] Spek, A. L. (2009). *Acta Cryst.* D**65**, 148–155. 10.1107/S090744490804362X PMC2631630 19171970

[bb7] Stoe & Cie (2009). *X-AREA* Stoe & Cie GmbH, Darmstadt, Germany.

